# 측정도구의 심리계량적 속성 3: 수렴, 판별, 집합 및 준거타당도

**DOI:** 10.4069/kjwhn.2021.08.18

**Published:** 2021-09-30

**Authors:** Eun-Hyun Lee

**Affiliations:** Graduate School of Public Health, Ajou University, Suwon, Korea; 아주대학교 보건대학원

**Keywords:** Convergent validity, Discriminant validity, Known-groups validity, Criterion validity, 수렴타당도, 판별타당도, 집합타당도, 준거타당도

## Abstract

Before evaluating convergent, discriminant, and known-groups validity, it is suggested to design an instrument that reflects hypothetical relationships or differences with other comparator instruments or groups. For criterion validity, a gold-standard instrument measuring the same construct should be carefully selected.

## 서론

이번 세번째 원고에서는 측정속성의 가설검정 구성타당도와 준거타당도에 대해 알아보고자 한다. 가설검정 구성타당도에서는 수렴, 판별, 및 집합타당도에 대해 알아볼 것이며, 준거타당도에서는 동시타당도와 예측타당도를 다룰 것이다.

## 가설검정 구성타당도(hypothesis-testing construct validity)

가설검정 구성타당도는 검정하고자 하는 도구로 측정한 점수와 어떤 다른 개념을 측정한 점수의 관계에 대해 연구자가 가설을 세운 후 자료 수집을 통해 수집된 자료를 분석한 결과가 미리 설정한 가설을 만족했는가를 보는 것이다. 이처럼 연구설계 단계에서 미리 가설을 가지고 시작해야 하므로 이를 강조하기 위해 “가설검정”이란 용어가 붙어 있다. 가설검정 구성타당도에는 수렴타당도(convergent validity), 판별타당도(discriminant or divergent validity), 집합타당도(known-groups validity)가 포함된다[1].

수렴타당도를 검정하기 위해서는 검정하고자 하는 도구가 측정하는 개념과 관련이 있다고 예상되는 유사개념을 먼저 선정해야 한다. 그리고 그 관련성이 어느 정도가 될지에 대한 크기와 방향성에 대한 가설을 가지고 자료수집과 분석을 통해서, 실증적으로 그런 결과가 나왔는지를 판단해야 한다. 예를 들어, Lee 등[2]은 당뇨병 헬스리터러시 측정도구(Diabetes Health Literacy Scale, DHLS)의 수렴타당도를 검정하기 위해 문헌 고찰을 하였다. 그리고 선행연구에서 헬스리터러시와 자기효능감은 실증적으로 중간 정도의 양의 관계가 있음을 확인하였다. 따라서 당뇨병 자가관리에 대한 자기효능감을 측정하는 Diabetes Management Self-Efficacy Scale (DMSES)를 수렴타당도의 비교측정도구(comparator instrument)로 결정하고, DHLS와 DMSES로 측정한 건강정보력과 자기효능감은 중간 정도의 양의 관계가 있을 것이라는 가설을 세웠다. 분석결과 DHLS와 DMSES는 *r*=.56 (*p*<.001)로 가설을 만족하였으므로 DHLS의 수렴타당도가 수립되었다고 하였다.

수렴타당도 검정에서 많은 연구자가 오류를 범하는 부분은, 수렴타당도 검정을 위해 사용하는 비교측정도구의 심리계량적 속성을 고려하지 않는 경우이다. 위의 DHLS 사례에서 수렴타당도 검정을 위해 사용된 DMSES는 한국 2형 당뇨병 환자를 대상으로 내용타당도, 요인구성타당도, 동시타당도, 내적일관성 및 검사-재검사 신뢰도가 충족된 도구이다[3]. 이것 외의 흔한 오류는, 수렴타당도를 검정하려는 도구와 비교측정도구 점수의 상관관계 정도와 방향성에 대한 가정 없이, 통계적으로 유의하게 나오면 무조건 수렴타당도를 만족하였다는 결론을 내리는 것이다[4]. 통계적으로 유의할 뿐 아니라 관계의 정도와 방향성을 만족해야 한다. 일반적으로 상관관계가 >.50이면 수렴타당도를 만족했다고 한다[5]. 하지만 임상적 특수성에 따라 이보다 약간 낮게 설정하는 경우도 있다. 예를 들어, 당뇨병 자가관리 측정도구의 수렴타당도 검증을 위해 생의학 지표(biomedical marker)인 당화혈색소(glycated hemoglobin, HbA1C)를 자주 사용한다. 하지만 실증적 선행연구에서 상관관계가 그리 높지 않게 보고되고 있으므로, 그 기준을 ≥.30으로 사용할 수 있다[6].

수렴타당도와 달리 판별타당도는 검정하고자 하는 측정도구점수가 이와 다른 속성을 측정하는 비교측정도구의 점수와 관련이 없거나 약한 상관관계(<.30)를 보일 것이라고 가설을 세운다[5]. 만약에 판별타당도에서 검정하고자 하는 측정도구와 다른 속성을 측정하는 도구의 점수 관계가 *r*=–.51 (*p*<.001)로 음의 관계가 나타났다고 하자. 이 사례에서는 상관관계가 유의할 뿐 아니라 중간 정도의 상관관계가 있는 것으로 나타나서 판별타당도를 만족했다고 보기 어렵다. 그런데도 간혹 어떤 연구자들은 판별타당도를 만족하였다는 잘못된 결론을 내리는 오류를 범한다.

집합타당도 검정을 위해서는 합리적 기준에 의해 연구대상자를 둘 이상의 하부 그룹으로 분류하고 이 그룹에 따라 관심개념 점수의 차이가 있을 것이라는 가설을 세운다. 그리고 실증적으로 하부 그룹에 따라 관심개념을 측정하는 도구로 측정한 점수에 차이가 있는지를 판단하는 것이다[7]. Lee 등[8]은 Arbuckle 등[9]이 개발한 당뇨병 환자의 증상 측정도구(Diabetes Symptom Checklist-Revised, DSC-R)의 집합타당도를 검정하기 위해 제2형 당뇨병이 있는 대상자의 HbA1c 수준을 연구 당시 당뇨병학회 기준에 의해 혈당이 조절된 집단과 그렇지 않은 집단으로 분류하고, 조절된 집단의 증상 점수가 낮을 것이라는 가설을 세웠다. 분석 결과, 혈당이 조절된 그룹의 DSC-R 점수가 유의하게 낮게 나타나서 집합타당도를 만족하였다고 보고하였다(*t*=−2.13, *p*=.03, *d*=0.65). 여기서 주의를 기울여야 할 점은 연구대상자를 하부 그룹으로 나누는 기준에 대한 합리적 근거가 있어야 한다는 것이다. 예를 들어, 간호사의 만족도 측정도구의 집합타당도를 검정한다고 연구대상자 점수를 상위 25%와 하위 25% 두 집단으로 나누어서 두 집단 만족도에 유의한 차이가 있었으므로 도구의 집합타당도가 만족하였다고 결론을 내려서는 안 된다. 왜냐하면, 상위 및 하위 25%로 나누어야 하는 근거가 없으며, 간호사의 상위 및 하위 25%의 점수는 연구마다 달라질 수 있기 때문이다.

일반적으로 수렴, 판별 및 집합타당도를 검정할 때 사용되는 비교측정도구로 대상자의 일반적 사항은 사용하지 말아야 한다. 예를 들어, 자기효능감 측정도구의 수렴 또는 집합타당도를 검정하기 위해서 논리적인 이유 없이 모든 연구의 데이터에 포함된 대상자의 일반적 특성(나이나 성별 등)을 사용하지 않는다. 물론 어떤 경우에는 나이나 성별을 중요한 비교 측정 개념으로 사용할 수 있다. 이런 경우에는 연구방법 단계에서 사용하고자 하는 일반적 특성 관련 변수를 타당도 검정에 필요한 관련 변수로 포함해서 그 근거를 제시해야 한다.

## 준거타당도(criterion validity)

준거(또는 기준)타당도는 검정하려는 도구가 측정하는 것과 같은 구성개념을 측정하는 표준화된(gold standard) 측정도구를 사용해서 얻은 결과를 비교하는 것을 말한다[1]. 준거타당도에는 동시타당도(concurrent validity)와 예측타당도(predictive validity)가 있다. 검정하려는 측정도구와 표준화된 측정도구가 같은 시점에서 동시에 측정하는 경우를 동시타당도라고 한다. 반면, 표준화된 측정도구가 미래의 다른 시점에서 측정되는 경우를 예측타당도라고 한다.

준거타당도 검정과 관련해서 국내 간호학에서 흔히 발생하는 오류를 살펴보면 다음과 같다[4]. 첫 번째 오류는 표준화된 도구를 잘못 선정하는 것이다. 표준화된 도구는 연구자가 검정하려고 하는 도구가 측정하는 같은 구성개념을 측정하는 도구로, 이미 신뢰도와 타당도가 수립된 도구여야 한다. 하지만 다른 유사개념을 측정하는 도구를 표준화된 도구라고 사용하는 경우가 많다. 즉, 유사개념을 측정하는 도구를 사용해서 수렴타당도를 검정한 후, 준거타당도를 검정했다고 보고하는 사례이다. 두 번째는 연구의 필요성에서 어떤 개념을 측정하는 기존의 도구에 심리계량적 문제가 있어서 새로운 도구 개발이 필요하다고 주장하고 나서, 연구의 필요성에서 비판되었던 기존의 문제 도구를 준거타당도 검정을 위한 표준화된 측정도구로 사용하는 것이다. 다시 말해서 연구자가 어떤 도구가 표준화된 도구가 아니라고 인정한 후에 표준화된 도구처럼 사용하는, 논리가 맞지 않는 연구를 수행하는 사례이다. 세 번째 경우는 예측타당도를 검정하기 위해서는 종적인 연구설계가 수행되어야 함에도 횡단적 연구설계를 하는 사례이다. 이렇게 횡단적 연구 설계를 시행하고서 예측타당도를 검정했다는 연구결과가 간혹 보고되기도 한다. 네 번째는 통계분석에 관한 것이다. 준거타당도에서 사용된 표준화된 도구가 연속형이면, 상관관계 분석 결과 *r*≥.70이면 준거타당도가 충족되었다고 한다. 표준화된 도구가 이분형이면 area under the curve (AUC)로 분석해서 AUC≥.70를 만족하면 된다[5]. 하지만 어떤 연구자는 연속형인 표준화된 도구를 합리적 이유 없이 이분형으로 분리하고 AUC로 분석해서 보고하는 예도 있다.

사실 측정도구 개발 연구에서 준거타당도 검정을 실시하기란 쉽지 않다. 가장 큰 이유로는 표준화된 측정도구를 찾기 어렵기 때문이다. 하지만 몇몇 경우에는 준거타당도 검정을 위한 표준화된 도구를 찾아볼 수 있다. 이미 개발된 측정도구의 축약형(short version)을 만들 때, 축약형의 준거타당도 검정에 대한 표준화된 도구로서 원래의 도구(original version)가 사용될 수 있다[10]. 전문가의 결정도 준거타당도를 위한 표준화된 도구로 사용될 수 있다[7]. 예를 들어서 Diagnostic and Statistical Manual of Mental Disorders (DSM-V)를 사용한 전문가의 결정은 여러 정신과적 문제와 관련된 자가보고형 측정도구의 준거 지표로 사용될 수 있다. 이외에도 같은 개념을 측정하는 일반형 측정도구가 표준화된 도구로 사용될 수 있다[11]. 예를 들어, 질병 특이형 삶의 질 측정도구의 준거타당도를 검정하기 위해서, 표준화된 도구로 일반형 삶의 질 측정도구(예, SF-36 Health Survey)를 사용할 수 있다. 하지만 이 경우에는 상관관계가 보통 정도여야 한다. 상관관계가 낮으면 개발하는 특이형 도구가 삶의 질을 측정한다고 보기 어려우며, 높으면 특이형이 아니라 일반형 삶의 질 도구와 유사하다고 볼 수 있기 때문이다.

## 요약

수렴, 판별 및 집합타당도를 검정하기 위해서는 연구자가 선행연구 결과를 바탕으로 검증하고자 하는 도구와 비교측정도구로 측정한 점수의 상관관계의 정도와 방향에 대한 가설을 세우고 시행해야 한다. 분석결과가 사전에 기획하였던 가설을 만족하였을 때, 그 타당도가 만족하였다고 할 수 있다. 준거타당도는 검정하고자 하는 도구가 측정하는 개념과 같은 개념을 측정하는 표준화된 도구가 있는 경우에 실시한다.

## References

[1] Mokkink LB, Terwee CB, Patrick DL, Alonso J, Stratford PW, Knol DL (2010). The COSMIN study reached international consensus on taxonomy, terminology, and definitions of measurement properties for health-related patient-reported outcomes. J Clin Epidemiol.

[2] Lee EH, Lee YW, Lee KW, Nam M, Kim SH (2018). A new comprehensive diabetes health literacy scale: development and psychometric evaluation. Int J Nurs Stud.

[3] Lee EH, van der Bijl J, Shortridge-Baggett LM, Han SJ, Moon SH (2015). Psychometric properties of the diabetes management self-efficacy scale in Korean patients with type 2 diabetes. Int J Endocrinol.

[4] Lee EH, Kang EH, Kang HJ (2020). Evaluation of studies on the measurement properties of self-reported instruments. Asian Nurs Res (Korean Soc Nurs Sci).

[5] de Vet HCW, Terwee CB, Mokkink LB, Knol DL (2011). Measurement in medicine: a practical guide.

[6] Lee J, Lee EH, Chae D, Kim CJ (2020). Patient-reported outcome measures for diabetes self-care: a systematic review of measurement properties. Int J Nurs Stud.

[7] Polit DF, Yang FM (2015). Measurement and the measurement of change.

[8] Lee EH, Lee KW, Song R, Snoek FJ, Moon SH (2014). Psychometric evaluation of the Korean version of the Diabetes Symptom Checklist-Revised (DSC-R) for patients with type 2 diabetes. Health Qual Life Outcomes.

[9] Arbuckle RA, Humphrey L, Vardeva K, Arondekar B, Danten-Viala M, Scott JA, Snoek FJ (2009). Psychometric evaluation of the Diabetes Symptom Checklist-Revised (DSC-R)-a measure of symptom distress. Value Health.

[10] Mokkink LB, de Vet HC, Prinsen CA, Patrick DL, Alonso J, Bouter LM (2018). COSMIN risk of bias checklist for systematic reviews of patient-reported outcome measures. Qual Life Res.

[11] Lee EH, Kwon O, Hahm KB, Kim W, Kim JI, Cheung DY (2016). Irritable bowel syndrome-specific health-related quality of life instrument: development and psychometric evaluation. Health Qual Life Outcomes.

